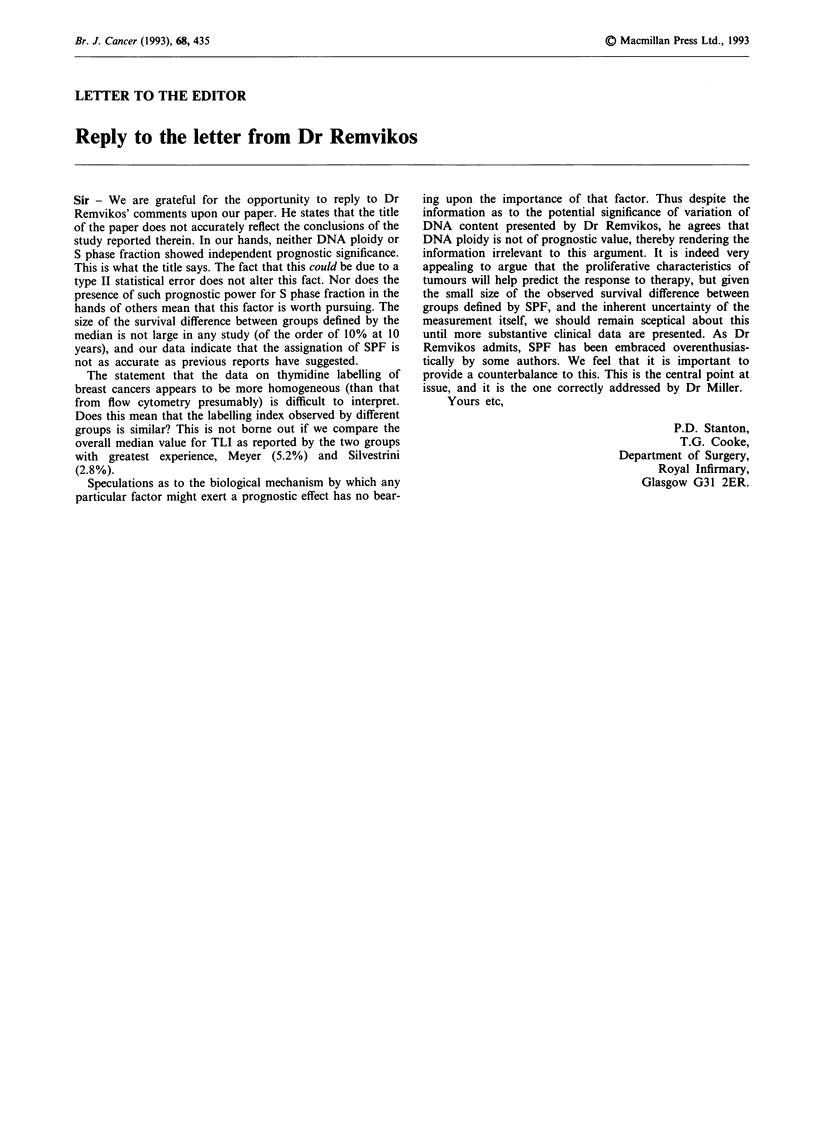# Reply to the letter from Dr Remvikos

**Published:** 1993-08

**Authors:** P.D. Stanton, T.G. Cooke


*Br. J. Cancer* (1993), **68**, 435 © Macmillan Press Ltd., 1993

LETTER TO THE EDITOR

### Reply to the letter from Dr Remvikos

**Sir** – We are grateful for the opportunity to reply to Dr Remvikos' comments upon our paper. He states that the title of the paper does not accurately reflect the conclusions of the study reported therein. In our hands, neither DNA ploidy or S phase fraction showed independent prognostic significance. This is what the title says. The fact that this *could* be due to a type II statistical error does not alter this fact. Nor does the presence of such prognostic power for S phase fraction in the hands of others mean that this factor is worth pursuing. The size of the survival difference between groups defined by the median is not large in any study (of the order of 10% at 10 years), and our data indicate that the assignation of SPF is not as accurate as previous reports have suggested.

The statement that the data on thymidine labelling of breast cancers appears to be more homogeneous (than that from flow cytometry presumably) is difficult to interpret. Does this mean that the labelling index observed by different groups is similar? This is not borne out if we compare the overall median value for TLI as reported by the two groups with greatest experience, Meyer (5.2%) and Silvestrini (2.8%).

Speculations as to the biological mechanism by which any particular factor might exert a prognostic effect has no bearing upon the importance of that factor. Thus despite the information as to the potential significance of variation of DNA content presented by Dr Remvikos, he agrees that DNA ploidy is not of prognostic value, thereby rendering the information irrelevant to this argument. It is indeed very appealing to argue that the proliferative characteristics of tumours will help predict the response to therapy, but given the small size of the observed survival difference between groups defined by SPF, and the inherent uncertainty of the measurement itself, we should remain sceptical about this until more substantive clinical data are presented. As Dr Remvikos admits, SPF has been embraced overenthusiastically by some authors. We feel that it is important to provide a counterbalance to this. This is the central point at issue, and it is the one correctly addressed by Dr Miller.

Yours etc,

P.D. Stanton,
T.G. Cooke,
Department of Surgery,
Royal Infirmary,
Glasgow G31 2ER.